# Plasma Lipidomic Features of Adolescent Major Depressive Disorder and Lipid Changes After Fluoxetine Treatment

**DOI:** 10.1002/cns.71202

**Published:** 2026-10-29

**Authors:** Chenxi Liu, Yao Li, Hanchen Hou, Jiamei Guo, Bingjie Qin, Xiaoxia Xu, Yuqian He, Tingting Lei, Fei Chen, Lige Zhang, Quanbing Liu, Li Gao, Jiaqi Shi, Haoran Xing, Teng Teng, Tian Qiu, Xinyu Zhou

**Affiliations:** ^1^ Department of Psychiatry, Key Laboratory of Major Brain Disease and Aging Research (Ministry of Education) The First Affiliated Hospital of Chongqing Medical University Chongqing China

**Keywords:** adolescent, fluoxetine, lipidomics, Major depressive disorder

## Abstract

**Background:**

Major depressive disorder (MDD) in adolescents is a major public health concern, yet objective biological markers and treatment‐related metabolic changes remain insufficiently understood. This study aimed to characterize plasma lipidomic alterations in adolescent MDD and explore lipid changes associated with fluoxetine treatment.

**Methods:**

This study enrolled 75 adolescents with MDD and 75 matched healthy controls (HCs). Among the adolescents with MDD, 44 had paired baseline and week‐8 plasma samples available and were included in the longitudinal analysis. Plasma lipidomic profiles were analyzed using ultra‐high‐performance liquid chromatography–tandem mass spectrometry (UHPLC–MS/MS).

**Results:**

A total of 865 lipid species were detected. Compared with HCs, adolescents with MDD showed distinct plasma lipidomic profiles, with 82 differentially abundant lipid species identified, primarily involving triglycerides, lysophospholipids, free fatty acids, membrane phospholipids, and sphingolipids. Among the machine‐learning models, logistic regression showed the highest discriminative performance, with a pooled out‐of‐fold AUC of 0.85 (95% CI, 0.79–0.91). Longitudinal analysis identified 21 lipid species meeting the differential criteria following fluoxetine treatment, mainly involving triglycerides and bile acid‐related metabolites. Integrative pathway and target analyses suggested that fluoxetine‐associated lipid changes may involve neurotransmitter signaling, inflammatory lipid metabolism, PI3K–Akt signaling, and nuclear receptor‐related pathways.

**Conclusion:**

This study characterizes alterations in plasma lipid metabolism in adolescent MDD and the lipidomic changes following fluoxetine treatment, providing new lipidomic evidence for candidate lipid signatures and potential treatment‐associated biological pathways.

**Trial Registration:**

ClinicalTrials.gov, NCT05814640. Registered 20 February 2023, https://clinicaltrials.gov/study/NCT05814640

## Graphical abstract

Dysregulated plasma lipid homeostasis with aberrant triglycerides, lysophospholipids, and membrane sphingolipids represents a core pathological feature of adolescent major depressive disorder. Eight‐week fluoxetine treatment remodels lipid profiles including bile acid metabolites and modulates key signaling pathways, supporting novel lipid‐based biomarker discovery and therapeutic target identification.

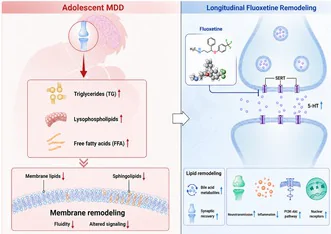

**Abbreviations**
AUC: Area under the receiver operating characteristic curve
BMI: Body mass index
CDRS‐R: Children's Depression Rating Scale‐Revised
DG: Diglyceride
DSM‐5: Diagnostic and Statistical Manual of Mental Disorders, Fifth Edition
ESI: Electrospray ionization
FC: Fold change
FDR: False discovery rate
FFA: Free fatty acid
GO: Gene Ontology
GPI: Glycosylphosphatidylinositol
HAMA: Hamilton Anxiety Rating Scale
HCs: Healthy controls
KEGG: Kyoto Encyclopedia of Genes and Genomes
KNN: k‐nearest neighbors
K‐SADS‐PL: Kiddie‐Schedule for Affective Disorders and Schizophrenia‐Present and Lifetime Version
LASSO: Least absolute shrinkage and selection operator
LPC: Lysophosphatidylcholine
LPE: Lysophosphatidylethanolamine
LR: Logistic regression
MDD: Major depressive disorder
MRM: Multiple reaction monitoring
NB: Naive Bayes
OPLS‐DA: Orthogonal partial least squares discriminant analysis
PC: Phosphatidylcholine
PCA: Principal component analysis
PE: Phosphatidylethanolamine
PPI: Protein–protein interaction
RF: Random forest
ROC: Receiver operating characteristic
SVM: Support vector machine
TG: Triglyceride
UHPLC–MS/MS: Ultra‐high‐performance liquid chromatography–tandem mass spectrometry
VIP: Variable importance in projection

## 1 Introduction

Major depressive disorder (MDD) during adolescence is a prevalent and disabling mental disorder characterized by persistent low mood, anhedonia, and marked impairment in physical and cognitive functions which severely disrupt patients' school performance and daily life [1]. MDD during adolescence contributes significantly to the global burden of disease, affecting approximately 4%–5% of the individuals in this age group [2]. Previous studies suggest that adolescents with MDD experience more severe social dysfunction [3] and a higher risk of suicide compared to adults [4]. Moreover, the distinctive biological and social characteristics of adolescence, including ongoing central nervous system development and hormonal changes, may contribute to atypical and heterogeneous presentations of MDD. These features make clinical diagnosis more challenging than in adults and underscore the need for objective diagnostic biomarkers [5, 6, 7]. In addition to diagnostic difficulties, the treatment of adolescent MDD remains clinically challenging. Current pharmacological regimens largely follow those used for adults, yet adolescents often show different and limited responses to existing antidepressants [8]. A previous network meta‐analysis identified fluoxetine as the only recommended medication for the treatment of adolescent MDD [9]. However, the efficacy of fluoxetine in real‐world clinical practice exhibits significant heterogeneity, and some patients fail to achieve an adequate response to the medication even after standardized treatment [10]. Therefore, objective and developmentally relevant biomarkers are needed not only to improve the early identification and stratification of adolescent MDD, but also to predict fluoxetine response and facilitate individualized pharmacotherapy.

Lipids play a central role in brain development, membrane structure, synaptic function, and inflammatory regulation. Polyunsaturated fatty acids (PUFAs) influence cell membrane fluidity and neurotransmission; phospholipids and sphingolipids are essential components of cellular membranes; and fatty acid derivatives participate in inflammatory signaling [7, 11, 12, 13]. Lipidomic studies have increasingly linked depression to broad disturbances in peripheral lipid metabolism [14]. In adult MDD, plasma and serum lipidomic studies have reported broad lipid metabolic alterations and identified lipid signatures with potential discriminatory value between patients with MDD and healthy controls [15, 16]. These findings provide a rationale for further examining whether similar or developmentally specific lipid alterations are present in adolescent MDD. Previous studies have suggested that adolescents with MDD exhibit peripheral lipid abnormalities, including reduced n‐3 PUFA levels, an increased n‐6/n‐3 PUFA ratio, enhanced lipid peroxidation, and disturbances in glycerophospholipid and sphingolipid metabolism [17, 18, 19]. These findings indicate that disruptions in lipid homeostasis may contribute to neurodevelopmental alterations, inflammatory activation, oxidative stress, and impaired membrane‐related signaling, which may represent important components of the pathophysiological mechanisms underlying adolescent MDD. With the rapid advancement of mass spectrometry, lipidomics has emerged as an important approach for biomarker discovery. Several studies have revealed lipid abnormalities in adolescents with MDD using different lipidomic platforms. Wang et al. conducted a systematic analysis of the erythrocyte membrane lipidome in adolescents with MDD and found that disruptions in membrane lipid homeostasis may contribute to the onset and progression of adolescent MDD [17, 18]. Gao et al. identified abnormalities in glycerophospholipids, glycerolipids, and sphingolipids in adolescents with MDD [20]. However, several questions in this field remain unresolved. First, previous studies have focused on erythrocyte membrane lipidomics and changes in membrane function, while research on the plasma lipidomic profiles and their changes in relation to disease status and treatment response has been relatively limited. Second, although lipid biomarkers have shown diagnostic potential in adolescent MDD, longitudinal treatment‐follow‐up studies are needed to clarify their association with treatment‐related biological changes.

To address these gaps, the present study conducted plasma lipidomic analysis in adolescents with MDD and matched healthy controls (HCs), with 8‐week fluoxetine treatment follow‐up in a subset of patients. We aimed to characterize disease‐related plasma lipidomic profiles, evaluate the classification potential of lipidomic features, and explore lipid changes associated with fluoxetine treatment and symptom improvement (Figure 1). Overall, this study may help clarify plasma lipidomic alterations in adolescent MDD and their changes following fluoxetine treatment.

**FIGURE 1:**
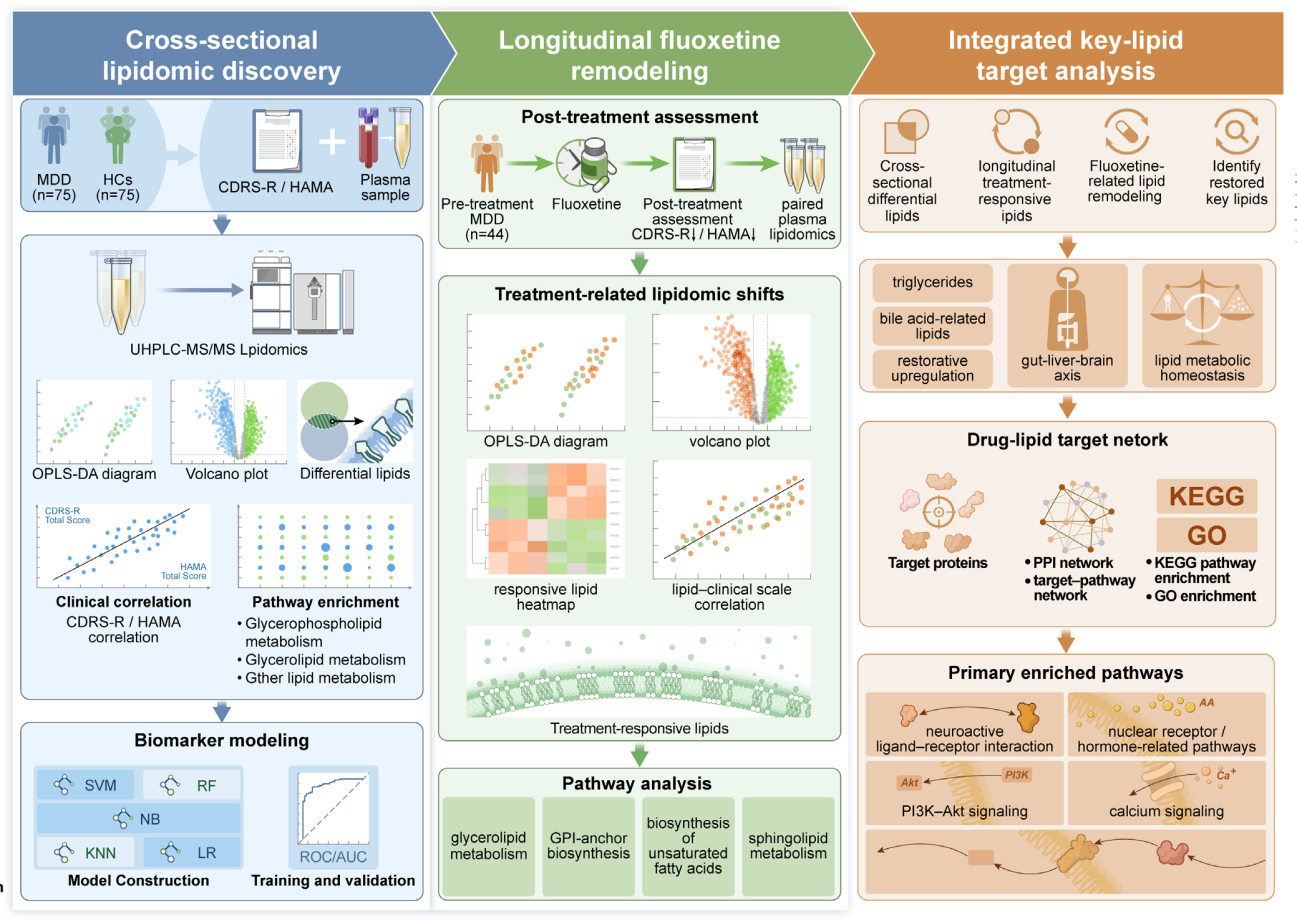
Schematic overview of the study design and integrated analytical workflow. The study comprises three core modules: Cross‐sectional plasma lipidomic profiling to identify MDD‐associated differential lipids, longitudinal characterization of lipid changes following fluoxetine treatment, and integrated bioinformatic analyses including biomarker modeling, pathway enrichment, and drug‐lipid target network construction.

## 2 Methods and Materials

### 2.1 Ethics Approval and Consent to Participate

This study was reviewed and approved by the Institutional Review Board (Ethics Committee) of the First Affiliated Hospital of Chongqing Medical University (Approval Nos. 2023–011‐02 and 20,225,402). Participants were recruited between May 2023 and January 2025. Written informed consent was obtained from each participant and at least one of the parents prior to study initiation. All procedures were carried out in accordance with the Declaration of Helsinki.

### 2.2 Study Participants

This study recruited 75 adolescents aged 12–18 years who met the diagnostic criteria for MDD according to the Diagnostic and Statistical Manual of Mental Disorders, Fifth Edition (DSM‐5). Diagnoses were assessed using the Kiddie‐Schedule for Affective Disorders and Schizophrenia‐Present and Lifetime Version (K‐SADS‐PL). Participants were required to have adequate hearing and vision to complete all study procedures. This requirement was confirmed during the initial clinical visit, based on participants' ability to answer questions fluently and engage in assessments conducted by trained researchers. Key exclusion criteria included: (1) a history of other severe psychiatric disorders or serious physical illnesses; (2) history of substance or medication abuse; (3) history of contraindications or intolerance to the investigational medication; (4) two or more failed trials of antidepressant medications; (5) insufficient washout from prior antidepressant treatment (< five drug half‐lives). Additional antidepressant or antipsychotic treatment was not permitted during the 8‐week follow‐up. Seventy‐five healthy adolescents matched for age, sex, and body mass index (BMI) were recruited as healthy controls from secondary schools in Chongqing, China (Jiulongpo District, Liangjiang New Area, Kaizhou District, and Dianjiang County). Among the 75 adolescents with MDD included in the cross‐sectional lipidomic cohort, 44 participants received oral fluoxetine at a dose of 20 mg/day for 8 weeks and had paired baseline and week‐8 plasma samples available; these participants were therefore included in the longitudinal lipidomic analysis. Paired week‐8 plasma samples were unavailable for the remaining 31 participants, who were therefore not included in the longitudinal lipidomic analysis (Figure S1). The severity of anxiety and depressive symptoms was assessed at each stage using the Children's Depression Rating Scale‐Revised (CDRS‐R) and Hamilton Anxiety Rating Scale (HAMA). Upon completion of all interviews, an independent inspector reviewed the recordings to ensure that every enrolled participant met the study eligibility criteria.

### 2.3 Sample Preparation

Baseline and follow‐up peripheral venous blood samples were collected under fasting conditions during the morning outpatient period (08:00–12:00). Peripheral venous blood (6 mL) was collected into labeled EDTA‐K2 tubes. A tourniquet was fixed 6 cm proximal to the puncture site, and repeated fist clenching dilated the antecubital vein. The skin was disinfected twice with 30 g/L iodine tincture and air‐dried before venipuncture; the needle entered the skin at 30° and advanced 10–15 mm into the vein at 5°. Post‐collection, the needle was withdrawn with 3 min of compression hemostasis. Specimens were mixed by gentle inversion 8–10 times and kept in an ice box temporarily.

After removal from the −80°C freezer, plasma samples were placed on ice and allowed to thaw slowly until no ice crystals remain. Vortex for 10 s, then a 50 μL aliquot was transferred to the corresponding centrifuge tube. Add 1 mL of lipid extraction solution containing the internal standard mixture (methyl tert‐butyl ether/methanol (3:1, v/v)) to the sample and vortex for 15 min to fully extract the lipids. Subsequently, add 200 μL of ultrapure water to induce phase separation, vortex for 1 min, and then centrifuge at 12,000 rpm for 10 min at 4°C. After centrifugation, quantitatively transfer 200 μL of the upper organic phase to a new centrifuge tube and evaporate to dryness using a centrifugal concentrator. Redissolve the dried lipid residue in 200 μL of the resuspension solution (acetonitrile/isopropanol (1:1, v/v)), vortex for 3 min to ensure thorough mixing, centrifuge again at 12,000 rpm for 3 min, and transfer the supernatant to a sample vial for subsequent instrumental analysis.

### 2.4 Ultra‐High‐Performance Liquid Chromatography–Tandem Mass Spectrometry (UHPLC–MS/MS) Analysis

Lipid separation and detection were performed using an ExionLC AD UHPLC system coupled with a QTRAP 6500+ triple quadrupole–linear ion trap mass spectrometer. A Thermo Accucore C30 column (2.6 μm, 2.1 mm × 100 mm) was used for chromatographic separation, with the column temperature maintained at 45 *C. mobile* phase A consisted of acetonitrile/water (60/40, v/v, containing 0.1% formic acid and 10 mmol/L ammonium formate), and mobile phase B consisted of acetonitrile/isopropanol (10/90, v/v, containing 0.1% formic acid and 10 mmol/L ammonium formate). The gradient elution program was set as follows: 0 min, A/B = 80:20; 2 min = 70:30; 4 min = 40:60; 9 min = 15:85; 14 min = 10:90; 15.5–17.3 min maintained at 5:95, and 17.5–20 min returned to 80:20. The flow rate was maintained at 0.35 mL/min, with an injection volume of 2 μL. Mass spectrometry acquisition utilized an electrospray ionization (ESI) source at 500°C; the ion spray voltage was set to 5500 V in positive ion mode and −4500 V in negative ion mode. The ion source gas 1 (GS1) was 45 psi; the ion source gas 2 (GS2) pressure was 55 psi, and the curtain gas pressure was 35 psi.

Lipid quantification was performed using multiple reaction monitoring (MRM) mode on a triple quadrupole mass spectrometer to specifically detect target lipids and their corresponding internal standards, thereby obtaining their respective chromatographic peak areas (A_x_ and AIS). The ratios of the integrated peak areas for all detected samples were substituted into a linear equation for calculation; after further substitution into the calculation formula, final lipid concentrations in the original plasma samples were calculated.

### 2.5 Analytical Quality Control and Data Preprocessing

Analytical quality control was performed throughout the LC–MS/MS analytical sequence using pooled quality‐control (QC) samples and blank samples. Technical replicates were performed for the pooled QC samples through repeated QC injections to assess analytical reproducibility and instrument stability. During data acquisition, one blank sample followed by one pooled QC sample was inserted after approximately every 10 study samples. Lipid species with poor analytical reproducibility were excluded, and only those with a coefficient of variation (CV) < 30% across pooled QC samples were retained for subsequent analyses. Instrument stability and potential signal drift were assessed by overlaying the total ion chromatograms (TICs) of repeatedly injected QC samples, which showed good overall overlap throughout the analytical sequences. Samples were analyzed in two analytical batches, and potential batch effects were corrected using the ComBat method before downstream statistical analyses. Missing values and values below the limit of detection were imputed as zero during data preprocessing. The complete injection sequences and QC TIC overlays are provided in the supplementary QC files.

### 2.6 Statistical Analysis

Lipidomic data were preprocessed and organized prior to statistical analysis, and potential batch effects arising from the two analytical batches were corrected using the ComBat method. For differential lipid analysis, the limma empirical Bayes linear modeling framework was used for the cross‐sectional comparison between the MDD and HC groups, whereas paired limma analysis and fold‐change calculation were used for the pre‐treatment and post‐treatment comparison, accounting for the within‐subject study design. For demographic and clinical variables, categorical variables were compared using the chi‐square test, between‐group continuous variables were compared using the independent‐samples *t*‐test, and pre‐treatment versus post‐treatment clinical scores were compared using the paired‐samples *t*‐test. Fold changes were calculated to quantify the magnitude and direction of lipid alterations. Differential lipids were identified using the combined criteria of *p* < 0.05, VIP ≥ 1, and |FC| > 1.2, with the *p*‐value criterion based on raw *p* values. Benjamini–Hochberg FDR‐adjusted *p* values were also calculated across all lipid species and reported. As a sensitivity analysis, the cross‐sectional differential lipid analysis was repeated with adjustment for smoking and drinking status using the same criteria. Principal component analysis (PCA) and orthogonal partial least squares discriminant analysis (OPLS‐DA) were then performed to evaluate global lipidomic patterns and group separation. For the cross‐sectional analysis, OPLS‐DA was performed using MetaboAnalystR (version 1.0.1) with one predictive component and one orthogonal component, and model validity was evaluated using seven‐fold cross‐validation and 200 permutation tests. For the longitudinal analysis, a paired multilevel OPLS‐DA was performed following within‐subject decomposition to account for the paired study design, with one predictive component and one orthogonal component. Model validity was further assessed using 200 pair‐restricted permutation tests, in which pre‐ and post‐treatment labels were permuted only within each participant. Within‐subject changes in treatment‐responsive lipids (Δlipid) were correlated with changes in CDRS‐R scores (ΔCDRS‐R) using Spearman correlation, and Δlipid was further compared between responders and non‐responders, defined as a ≥ 50% reduction in CDRS‐R score from baseline.

Pathway enrichment analysis was performed in MetaboAnalyst 5.0 using the *Homo sapiens* KEGG pathway library, with the hypergeometric test for enrichment and relative‐betweenness centrality for pathway topology. To evaluate the classification potential of lipidomic features, several supervised machine‐learning models were constructed based on the identified differential lipids, including support vector machine (SVM), random forest (RF), naive Bayes (NB), k‐nearest neighbors (KNN), and logistic regression (LR). Machine‐learning analyses were performed in R 4.3.1 using caret (v7.0–1), pROC (v1.19.0.1), glmnet (v4.1–10), randomForest (v4.7–1.2), kernlab (v0.9–33), and klaR (v1.7–4), with a fixed random seed of 2026. Model performance was evaluated using nested stratified five‐fold cross‐validation. Within each outer training fold, data preprocessing, feature scaling, differential‐lipid screening, and RF/LASSO feature selection were performed. LASSO used *α* = 1 and λ1se, RF used 1000 trees and the top 20 features, and hyperparameters were tuned by three‐fold cross‐validation. The area under the ROC curve (AUC) was calculated to compare model performance. Permutation testing was additionally performed by randomly shuffling the class labels and repeating the complete cross‐validation procedure, and the observed model performance was compared with the permutation‐derived null distribution. For drug–lipid target analysis, overlapping cross‐sectional and longitudinal lipids with opposite changes were selected. Targets were predicted using PubChem and SwissTargetPrediction, followed by STRING/Cytoscape network analysis and GO/KEGG enrichment using clusterProfiler and pathview. All statistical tests were two‐sided, and *p* < 0.05 was considered statistically significant. Statistical analyses were performed using R software version 4.3.1.

## 3 Results

### 3.1 Demographic and Clinical Characteristics

A total of 75 healthy adolescents and 75 adolescents with MDD were included in this study (Table 1). No significant differences were observed between the HCs and MDD groups in sex, age, or body mass index (BMI) (all *p* > 0.05). Compared with the HCs group, adolescents with MDD had significantly higher CDRS‐R scores (61.25 ± 13.27 vs. 24.65 ± 8.23, *p* < 0.01) and HAMA scores (16.87 ± 4.26 vs. 2.65 ± 1.81, *p* < 0.01), indicating greater depressive and anxiety symptom severity in the MDD group (Table 1). Within the MDD cohort, no significant baseline differences in sex, age, BMI, CDRS‐R, or HAMA scores were observed between the Longitudinally included and Longitudinally non‐included subgroups (all *p* > 0.05) (Table S1).

**TABLE 1:** Demographics and clinical characteristics in HCs and MDD.

	HCs	MDD	*p*
*N*	75	75	
Sex (Female, Percentage)^a^	45 (60.0)	54 (72.0)	0.17
Age (years, mean ± SD)^b^	15.58 ± 1.70	15.59 ± 1.57	0.49
BMI (kg/m^2^, mean ± SD)^b^	20.64 ± 3.56	20.57 ± 2.66	0.90
CDRS‐R (mean ± SD)^b^	24.65 ± 8.23	61.25 ± 13.27	< 0.01
HAMA (mean ± SD)^b^	2.65 ± 1.81	16.87 ± 4.26	< 0.01

*Note:* Continuous variables are presented as mean ± standard deviation (SD).

Abbreviations: BMI, Body Mass Index; CDRS‐R, Children's Depression Rating Scale‐Revised; HAMA, Hamilton Anxiety Rating Scale.

^a^ Chi‐Square test.

^b^ *t*‐test.

### 3.2 Lipid Class Distribution and Multivariate Analysis

A total of 865 lipid species were detected in plasma samples from 75 healthy adolescents and 75 adolescents with MDD. At the broad lipid‐category level, glycerophospholipids accounted for the largest proportion of the detected lipid species (GP, 42.43%), followed by glycerolipids (GL, 31.10%) and sphingolipids (SP, 18.53%) (Figure S2). At the lipid‐subclass level, triglycerides represented the largest subgroup (TG, *n* = 251, 29.02%), followed by phosphatidylethanolamine and ether‐linked phosphatidylethanolamine species (PE/PE‐O, *n* = 123, 14.22%) and ceramides (Cer‐NS, *n* = 46, 5.32%) (Figure 2A and Figure S2). Unsupervised principal component analysis (PCA) showed partial separation between the HCs and MDD groups (Figure 2B). The OPLS‐DA score plot further demonstrated apparent separation between the two groups in the supervised discriminant model (Figure 2C). The model yielded R^2^X = 0.359, R^2^Y = 0.965, and Q^2^ = 0.507. In 200 permutation tests, none of the permuted models reached the observed model performance (0/200, *p* < 0.005) (Figure 2D).

**FIGURE 2:**
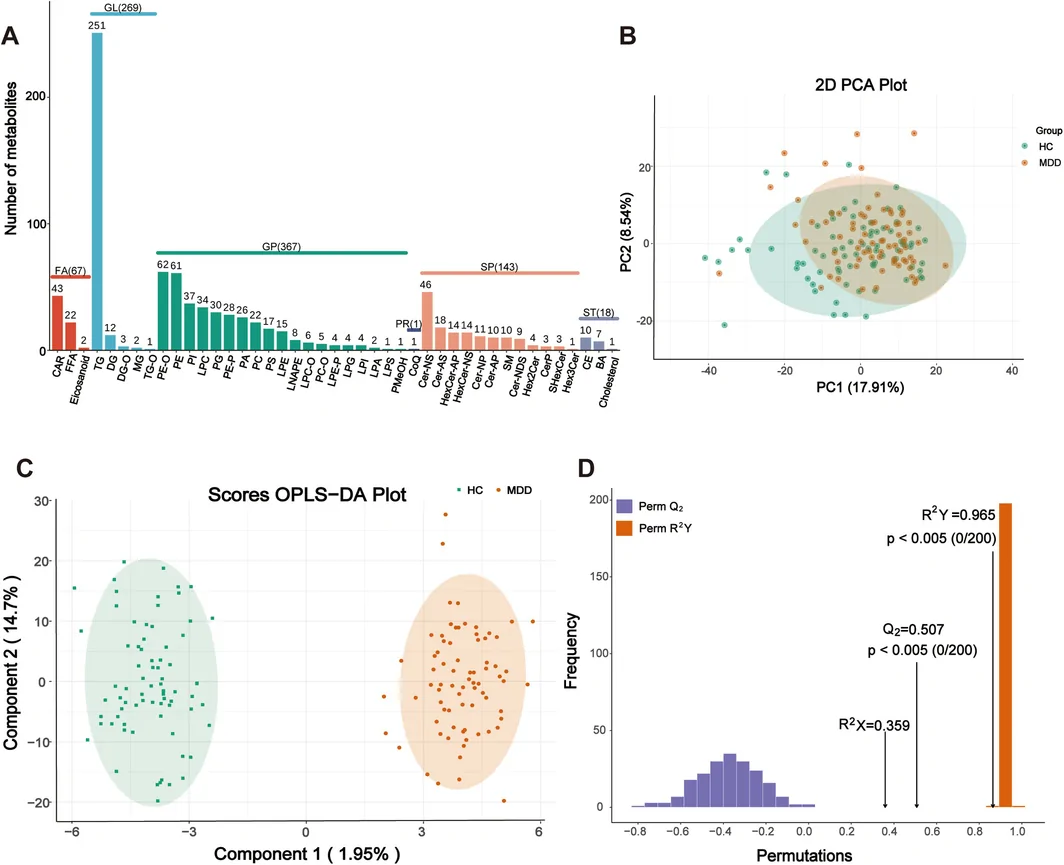
Plasma lipidomic profiles, PCA, and OPLS‐DA analysis of the HCs and MDD groups. (A) Secondary subclass distribution of detected plasma lipid species in the study cohort; (B) Two‐dimensional PCA score plot showing the overall distribution of plasma lipid profiles in the HCs and MDD groups; (C) Supervised OPLS‐DA score plot showing the distribution of the HCs and MDD groups in the model component space; (D) Permutation test plot of the OPLS‐DA model evaluating model fit and predictive stability.

### 3.3 Differential Lipid Analysis and Structural Characterization

A total of 82 significantly differentially abundant lipid species were identified between the HCs and MDD groups (*p* < 0.05, |FC| > 1.2, and VIP ≥ 1). Among these, 26 lipid species remained significant after FDR correction (FDR‐adjusted *p* values < 0.05). Compared with the HCs group, 14 lipid species were increased, and 68 were decreased in the MDD group (Figure 3A). Sensitivity analysis adjusting for smoking and drinking identified 83 differential lipids, 79 of which overlapped with the primary 82 (96.3%), with concordant directions and highly correlated effect sizes (*r* = 0.9997). No significant baseline lipidomic differences remained between the longitudinally included and non‐included subgroups after FDR correction (Table S4). The clustering heatmap of the top 25 differentially abundant lipid species showed an overall reduction in lipid abundance in the MDD group (Figure 3B). To further characterize the structural features of these differential lipids, we analyzed their total carbon number and degree of unsaturation. The distribution of total carbon number showed two major peaks. Lipids with C46–C55 total carbon atoms accounted for 49.8% of the differential lipids, representing the predominant peak, whereas lipids with 21–25 total carbon atoms accounted for 15.8%, representing the secondary peak (Figure 3C). The distribution of unsaturation was mainly concentrated in lipids containing 0–7 double bonds. Among lipids with more than 7 double bonds, significantly differentially abundant lipid species were limited to those containing 10 double bonds, which accounted for the highest proportion among all unsaturation categories, reaching 16.7% (Figure 3D).

**FIGURE 3:**
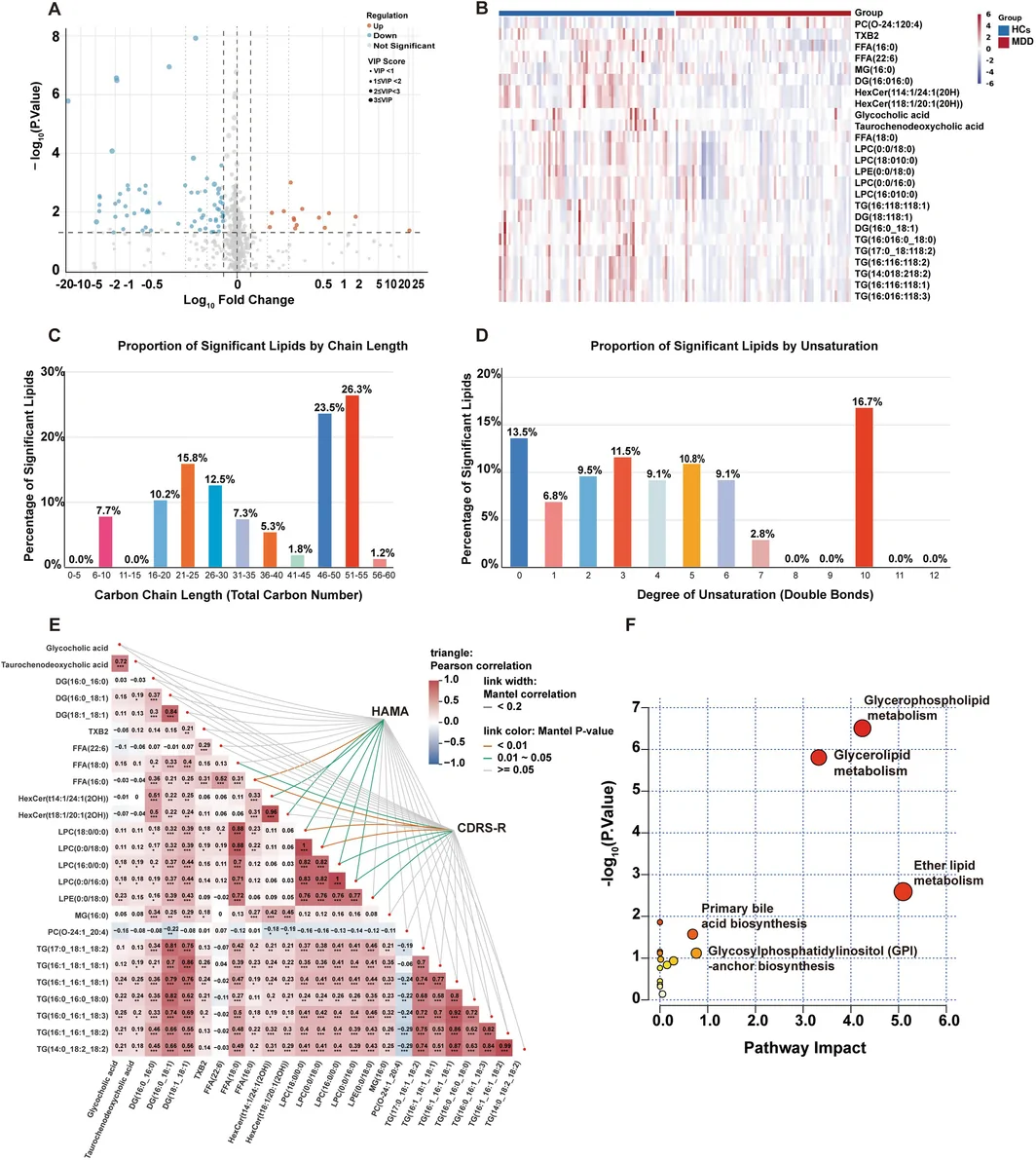
Differential lipid profiles between HCs and MDD groups and pathway enrichment analysis. (A) Differential lipid profiling between HCs and MDD groups; (B) Heatmap of the top 25 differential lipid species between HCs and MDD groups; (C) Characteristics of the carbon chain length distribution of differentially abundant lipid species; (D) Characteristics of the unsaturation distribution of differentially abundant lipid species; (E) Pearson correlation analysis among differentially abundant lipid species and Mantel associations between lipid profiles and CDRS‐R/HAMA scores; (F) Pathway enrichment analysis of differential lipids in adolescent MDD.

### 3.4 Correlation Analysis and Pathway Enrichment of Differential Lipids

Pearson correlation analysis was used to assess pairwise correlations among the top 25 differentially abundant lipid species. Mantel tests were further performed to evaluate associations between the lipid abundance distance matrix and clinical symptom‐score distance matrices for CDRS‐R and HAMA. Pearson correlation analysis revealed two major lipid correlation modules. Within the triglyceride (TG) module, TG (16:0_16:1_18:3), TG (14:0_18:2_18:2), and TG (16:1_16:1_18:2) were positively correlated with each other (*r* = 0.55–0.86), suggesting coordinated alterations in triglyceride‐related metabolism (Figure 3E). Strong positive correlations were also observed among lysophosphatidylcholine (LPC) and lysophosphatidylethanolamine (LPE) species (*r* = 0.72–0.99), indicating coordinated remodeling of the lysophospholipid pool (Figure 3E). Clinical association analysis showed that CDRS‐R scores were significantly associated with FFA (18:0), FFA (16:0), and LPC/LPE species (*p* < 0.02), whereas HAMA scores were significantly associated with FFA (16:0), HexCer species, and LPC/LPE species (*p* < 0.05) (Figure 3E). Lipidomics pathway enrichment analysis revealed that differentially abundant lipid species were mainly enriched in the glycerophospholipid metabolism, glycerolipid metabolism, and ether lipid metabolism pathways (Figure 3F). Among these pathways, glycerophospholipid metabolism (FDR = 2.490 × 10^−5^, Impact = 0.424) and glycerolipid metabolism (FDR = 6.253 × 10^−5^, Impact = 0.333) exhibited high statistical significance, while ether lipid metabolism had a high pathway impact score (FDR = 0.068, Impact = 0.509), suggesting that membrane lipid remodeling, abnormal glycerolipid metabolism, and ether lipid‐related metabolic disorders may be key features of lipid metabolic changes in this study population. Primary bile acid biosynthesis (FDR = 0.427, Impact = 0.068) and glycosylphosphatidylinositol (GPI)‐anchor biosynthesis (FDR = 0.762, Impact = 0.076) also showed modest enrichment, although their statistical significance and pathway impact were relatively limited (Figure 3F).

### 3.5 Machine Learning‐Based Classification Using Differential Lipids

To evaluate the classification potential of plasma lipidomic features in adolescent MDD, random forest and LASSO regression were used as complementary feature‐selection methods. Random forest variable importance analysis identified several highly informative lipid features, with FFA(16:0), HexCer(t18:1/20:1(2OH)), HexCer(t14:1/24:1(2OH)), HexCer(t14:1/17:0(2OH)), and LPC(0:0/18:0) ranking among the most important predictors (Figure 4A). In parallel, LASSO regression selected lipid variables with non‐zero coefficients, reflecting their contribution to the penalized classification model (Figure 4B). Random forest selected 20 candidate features, of which 10 overlapped with those identified by LASSO (Figure 4C). For classification analysis, feature screening and RF/LASSO‐based feature selection were performed within each training fold of the nested stratified five‐fold cross‐validation framework. Based on pooled out‐of‐fold validation predictions, LR showed the highest discriminative performance with an AUC of 0.85 (95% CI, 0.79–0.91), followed by RF (AUC = 0.84, 95% CI, 0.78–0.90), SVM (AUC = 0.82, 95% CI, 0.75–0.89), KNN (AUC = 0.79, 95% CI, 0.72–0.86), and NB (AUC = 0.79, 95% CI, 0.72–0.86) (Figure 4D). Sensitivity, specificity, and accuracy based on pooled out‐of‐fold predictions are summarized in Table S3. Permutation testing further showed that the observed classification performance exceeded that obtained under randomly permuted class labels. In the training folds, all five models demonstrated high classification performance, with AUC values exceeding 0.85 (Figure S3). These results indicate that the lipidomics‐based models achieved moderate to good discriminative performance in distinguishing adolescents with MDD from HCs.

**FIGURE 4:**
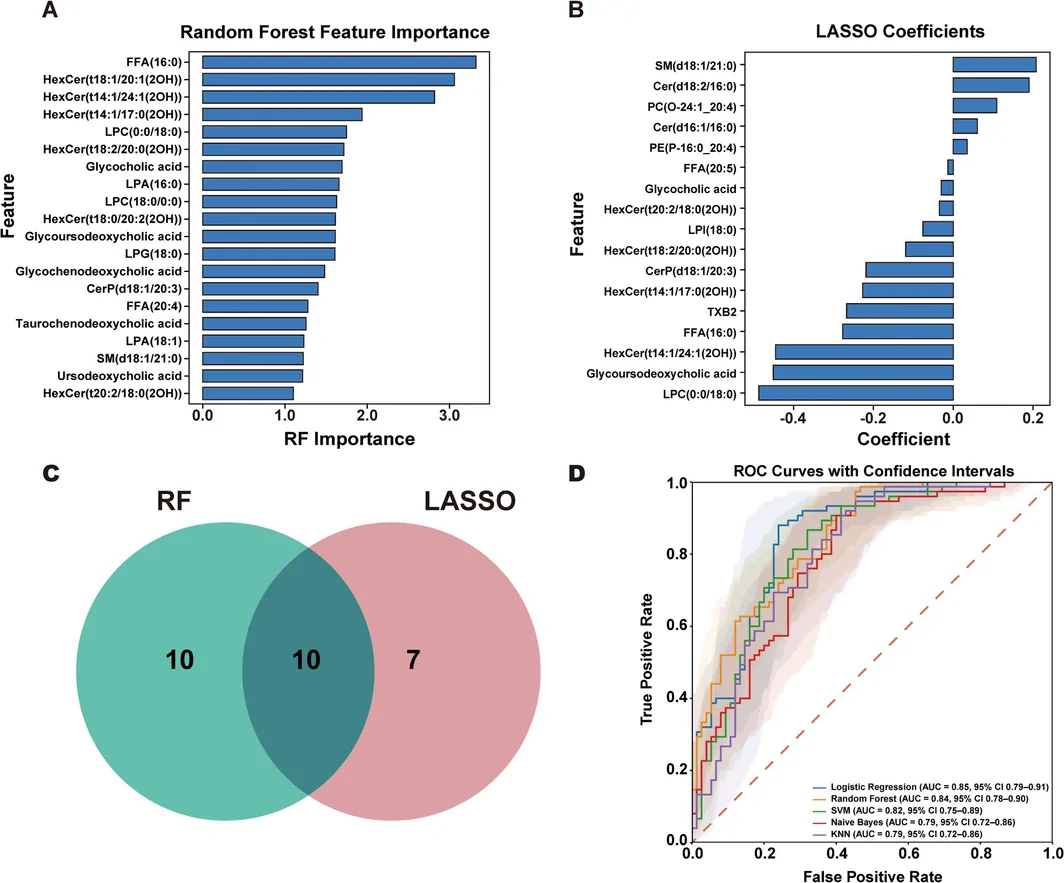
ROC curves for machine learning classification models based on lipidomic features. (A) Random Forest (RF) selects feature lipids based on differentially abundant lipid species; (B) LASSO regression selects feature lipids based on differentially abundant lipid species; (C) Venn diagram of feature lipids selected by RF and LASSO; (D) Receiver operating characteristic (ROC) curves with 95% confidence intervals for the five machine‐learning models based on pooled out‐of‐fold validation predictions.

### 3.6 Lipidomic Changes Before and After Fluoxetine Treatment

Among the adolescents with MDD, 44 participants with paired baseline and week‐8 plasma samples were included in the longitudinal analysis. After 8 weeks of fluoxetine treatment, CDRS‐R scores were significantly reduced from 63.84 ± 9.78 at baseline to 39.16 ± 6.42 after treatment (*p* < 0.01). HAMA scores also decreased significantly from 17.36 ± 4.44 to 14.70 ± 5.69 (*p* = 0.02), indicating improvement in anxiety symptoms (Table 2). The paired multilevel OPLS‐DA score plot showed visual separation between the pre‐treatment and post‐treatment samples in the model component space (Figure 5A). Pair‐restricted permutation testing with 200 permutations yielded an observed separation statistic of 0.679 and a permutation *p* value of 0.313 (Figure 5B). Differential lipidomic analysis identified 21 lipid species that changed significantly after fluoxetine treatment (*p* < 0.05, VIP ≥ 1, |FC| > 1.2). Among these, 9 lipid species were increased and 12 were decreased after treatment (Figure 5C). Among these, 8 lipid species remained significant after FDR correction (FDR‐adjusted *p* values < 0.05) (Table S2). The clustering heatmap further showed distinct abundance patterns for several treatment‐associated lipids, including increases in glycocholic acid, taurochenodeoxycholic acid, and several TG species, together with decreases in TxB3, FFA(10:0), FFA(16:0), DG(16:0_16:0), MG(16:0), and several HexCer species (Figure 5D).

**TABLE 2:** Clinical characteristics before and after fluoxetine treatment.

	Pre‐treat‐MDD	Post‐treat‐MDD	*p*
*N*	44	44	
CDRS‐R (mean ± SD)^a^	63.84 ± 9.78	39.16 ± 6.42	< 0.01
HAMA (mean ± SD)^a^	17.36 ± 4.44	14.70 ± 5.69	0.02

*Note:* Continuous variables are presented as mean ± standard deviation (SD).

Abbreviations: CDRS‐R, Children's Depression Rating Scale‐Revised; HAMA, Hamilton Anxiety Rating Scale.

^a^ Paired samples *t*‐test.

**FIGURE 5:**
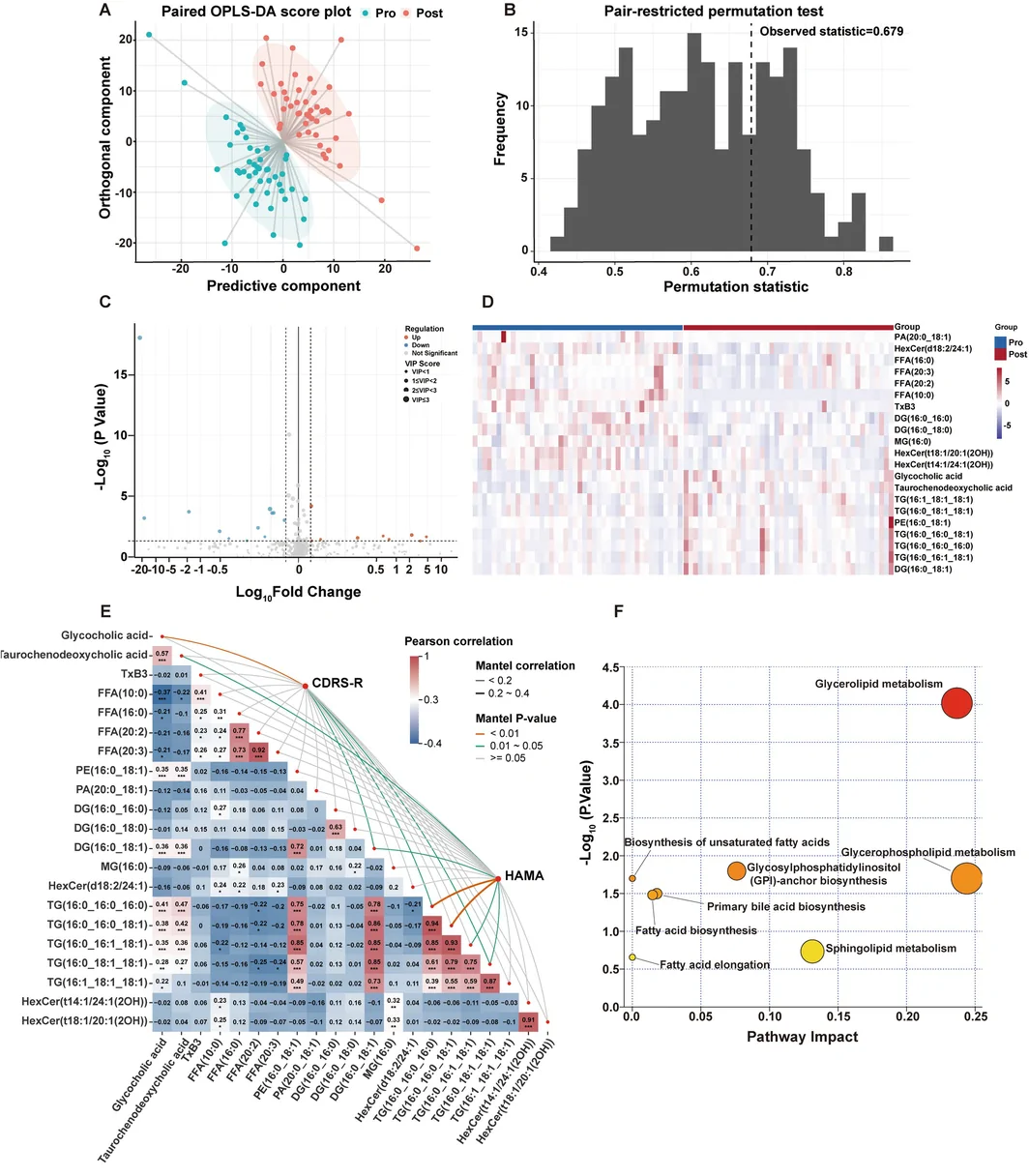
Lipidomic changes before and after fluoxetine treatment and pathway analysis. (A) Paired multilevel OPLS‐DA score plot showing the separation of the Pre‐treat‐MDD and Post‐treat‐MDD groups in the model component space; (B) Pair‐restricted permutation test of the longitudinal OPLS‐DA model based on 200 permutations; (C) Volcano plot of differential lipids between post‐treatment and pre‐treatment time‐points; (D) Heatmap of the top treatment‐responsive lipids; (E) Pearson correlation analysis among treatment‐responsive lipid species and Mantel associations with clinical scores; (F) Analysis of lipid pathways in pre‐treat‐MDD and post‐treat‐MDD groups.

Pearson correlation analysis was performed to assess pairwise relationships among treatment‐responsive lipid species, and Mantel tests were used to evaluate their associations with clinical symptom scores. A fatty‐acid‐related correlation cluster was observed, in which FFA(20:2) was strongly positively correlated with FFA(20:3) (*r* = 0.92), while FFA(16:0) was positively correlated with FFA(20:2) (*r* = 0.77) and FFA(20:3) (*r* = 0.73). A triglyceride‐related correlation cluster was also identified. TG(16:0_16:0_16:0) was strongly positively correlated with TG(16:0_16:0_18:1) (*r* = 0.94), and TG(16:0_16:0_18:1) was positively correlated with TG(16:0_16:1_18:1) (*r* = 0.93) (Figure 5E). CDRS‐R scores were significantly associated with glycocholic acid (*p* = 0.008), DG(16:0_18:1) (*p* = 0.038), TG(16:0_16:0_16:0) (*p* = 0.048), and TG(16:0_18:1_18:1) (*p* = 0.027), whereas HAMA scores were significantly associated with taurochenodeoxycholic acid (*p* = 0.026), DG(16:0_18:1) (*p* = 0.025), TG(16:0_16:0_16:0) (*p* = 0.009), TG(16:0_16:0_18:1) (*p* = 0.006), TG(16:0_16:1_18:1) (*p* = 0.026), and TG(16:0_18:1_18:1) (*p* = 0.040) (Figure 5E). Exploratory analyses showed that ΔHexCer(d18:2/24:1) was associated with ΔCDRS‐R (*p* = 0.008) (Figure S4), while ΔHexCer(d18:2/24:1) and ΔMG(16:0) differed between responders and non‐responders (*p* = 0.004 and *p* = 0.023, respectively) (Figure S5). The pathway topology bubble plot indicates that glycerolipid metabolism showed the strongest enrichment significance (−log_10_[*p*] = 4.02) with a high pathway impact (Impact = 0.237), while glycerophospholipid metabolism had the highest pathway impact (Impact = 0.244, −log_10_[p] = 1.70), followed by GPI‐anchor biosynthesis (Impact = 0.076, −log_10_[*p*] = 1.80), whereas biosynthesis of unsaturated fatty acids, primary bile acid biosynthesis, fatty acid biosynthesis, and sphingolipid metabolism were also implicated in the treatment‐associated lipidomic changes (Figure 5F).

### 3.7 Drug–Target and Pathway Enrichment Analysis of Fluoxetine‐Associated Lipid Targets

By integrating fluoxetine‐related targets with key lipid‐associated targets, a total of 153 candidate drug‐target proteins were identified. A protein–protein interaction (PPI) network was constructed using STRING analysis. The results indicated that nodes such as TNF, NCOR1, RXRA, PTGS2, SLC6A3, MAOA, AR, SLC6A4, PRKCA, PPARG, PIK3CA, CYP19A1, and PPARA were located in the relatively central region (Figure 6A). Gene Ontology (GO) enrichment analysis results suggested that these target proteins were primarily concentrated in biological processes related to G protein‐coupled receptor signaling, monoamine transport, signal transduction, cytoplasmic calcium ion concentration regulation, cellular calcium ion homeostasis, and blood pressure regulation. These proteins were mainly localized to synaptic membranes, presynaptic membranes, postsynaptic membranes, membrane rafts, and membrane microdomains. In terms of molecular function, these proteins were enriched in neurotransmitter receptor activity, serotonin receptor activity, catecholamine binding, and prostaglandin receptor activity (Figure 6B). The target–pathway network diagram further illustrated the contributions of different targets to the enriched Kyoto Encyclopedia of Genes and Genomes (KEGG) pathways. Neurotransmitter receptor‐related proteins, such as HTR1A, DRD2, ADRA2A, CHRM1, and CNR1, were linked to pathways associated with neuroactive ligands. Lipid metabolism and inflammatory mediator‐related targets, such as PTGS2, ALOX5, LTA4H, PLA2G2A, and PLA2G5, were primarily involved in the arachidonic acid metabolism pathway. Furthermore, targets such as PIK3CA, MTOR, GSK3B, BCL2L1, XIAP, MDM2, RXRA, and NCOR1 were associated with the PI3K‐Akt signaling pathway, while targets including AR, ESR2, PGR, NR3C1, THRA, THRB, PPARA, RXRA, and VDR were involved in hormone and thyroid hormone‐related signaling pathways (Figure 6C). KEGG analysis indicated that pathways such as neuroactive ligand–receptor interactions, the PI3K‐Akt signaling pathway, calcium signaling pathways, and arachidonic acid metabolism are notably enriched. These enrichment results indicated that the predicted targets were associated with neurotransmitter signaling, inflammatory lipid metabolism, PI3K–Akt signaling, and endocrine‐related pathways (Figure 6D). Collectively, these findings suggest that fluoxetine‐associated lipid targets may involve neurotransmitter, inflammatory, calcium, PI3K–Akt, and nuclear receptor‐related pathways.

**FIGURE 6:**
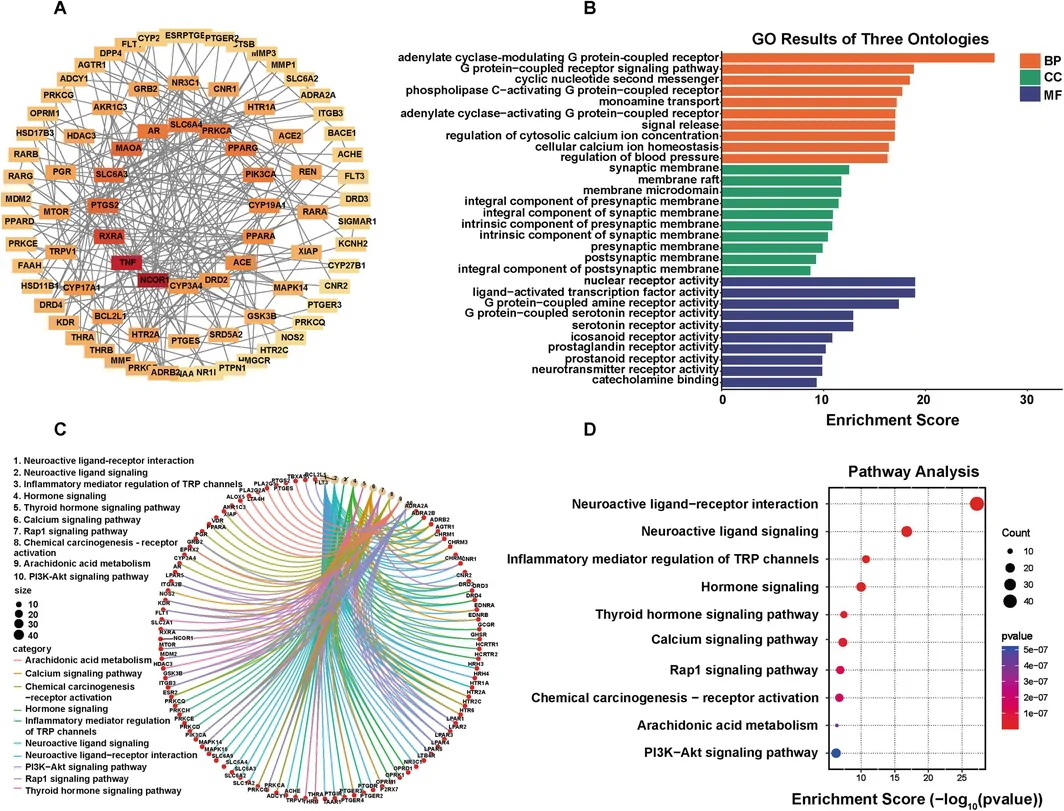
Network and enrichment analysis of drug‐binding proteins targeted by fluoxetine and key lipid species. (A) PPI network analysis; (B) GO functional enrichment analysis results; (C) Target‐pathway association network diagram; (D) Pathway enrichment bubble chart for drug‐targeted binding proteins.

## 4 Discussion

This study demonstrated marked alterations in plasma lipid metabolism in adolescents with MDD and identified lipidomic signatures with potential diagnostic and treatment‐related relevance. Compared with healthy controls, adolescents with MDD showed distinct plasma lipidomic profiles and 82 differentially abundant lipid species. Pathway analyses suggested that these alterations were primarily related to glycerolipid metabolism, glycerophospholipid metabolism, ether lipid metabolism, and membrane lipid remodeling. Lipidomics‐based machine learning models further showed moderate to good discriminative performance, indicating the potential utility of plasma lipid features as discriminative lipid features for adolescent MDD. After 8 weeks of fluoxetine treatment, clinical symptoms improved significantly, accompanied by changes in 21 treatment‐responsive lipid species. Integrated target and pathway analyses indicated that fluoxetine‐associated lipid changes may involve neurotransmitter receptor signaling, inflammatory lipid mediator metabolism, PI3K–Akt signaling, and nuclear receptor‐related pathways. Overall, these findings suggest that peripheral lipid dysregulation may be involved in adolescent MDD and may reflect biological changes associated with fluoxetine response.

The study found that adolescents with MDD showed decreased plasma levels of DG, LPC, FFA, and TG, together with increased levels of PC and PE. These changes suggest that adolescent MDD may involve coordinated disturbances in glycerolipid metabolism, lysophospholipid metabolism, and membrane phospholipid remodeling. Given the roles of TG and DG in lipid storage and energy metabolism, their reduction may reflect altered energy homeostasis in the depressive state [21, 22]. The reduced levels of several FFA‐related lipids may reflect altered polyunsaturated fatty acid metabolism. Given the role of omega‐3 polyunsaturated fatty acids in membrane fluidity, organelle function, and synaptic transmission, these changes may be associated with lipid‐related mechanisms underlying depressive symptoms [23]. Research on free fatty acids in depression has progressed from animal studies to randomized controlled trials in clinical populations, with particular attention to omega‐3 polyunsaturated fatty acids [24, 25, 26, 27, 28]. However, the reported effects remain heterogeneous and may differ between adolescents and adults, possibly due to differences in fatty acid composition, developmental stage, and neurobiological context [24, 25, 26, 27, 28]. LPC is generated from phosphatidylcholine (PC) via phospholipase A2 and is involved in membrane remodeling and inflammatory signaling [29, 30, 31]. PC and PE are major structural phospholipids that contribute to membrane integrity and curvature [32]. In this study, LPC levels were decreased in the MDD group, whereas PC and PE levels were increased, suggesting alterations in membrane phospholipid homeostasis. These changes may reflect dysregulated membrane remodeling processes in adolescent MDD and are consistent with previous lipidomic studies reporting associations between phospholipid metabolism and depressive symptoms in adolescent populations [21]. Analysis of carbon chain length and degree of unsaturation revealed a characteristic distribution pattern among differentially abundant lipid species. A major peak was observed in the C46–C55 range, which is consistent with the structural properties of polyacylated lipids such as triglycerides (TG) and diglycerides (DG). This finding aligns with the observed coordinated correlations among TG‐ and DG‐related species, suggesting that these changes may reflect lipid remodeling within energy storage and glycerolipid metabolic pathways. A second peak was identified in the C21–C25 range, which may include medium‐ and long‐chain fatty acids and selected sphingolipid‐related species [12]. These lipid species are closely related to membrane phospholipid composition and signaling lipid metabolism, indicating that MDD‐associated lipid alterations may involve coordinated changes in both energy‐related and membrane‐related lipid pools. Five machine learning models (SVM, LR, RF, NB, and KNN) were constructed to evaluate the diagnostic performance of lipidomic features for adolescent MDD. All models showed moderate discriminative ability in both training and testing sets, with SVM, RF, and LR performing relatively better. These results suggest that plasma lipidomic profiles may have potential as candidate lipid signatures for adolescent MDD. It is worth noting that bile acid metabolites, including glycocholic acid and taurochenodeoxycholic acid, were identified among the selected features, suggesting a potential involvement of bile acid metabolism in adolescent MDD [33, 34]. These findings support the potential utility of plasma lipidomic features for adolescent MDD. However, independent cohort validation is required to confirm the stability and clinical applicability of these findings.

Previous studies have suggested that fluoxetine may influence lipid metabolism in addition to its classical action on the serotonin transporter [35, 36]. In this study, 21 lipid species changed significantly after 8 weeks of fluoxetine treatment. By integrating these treatment‐associated lipids with the cross‐sectional MDD‐related lipid profile, we identified 9 lipid species, mainly triglycerides and bile acid‐related metabolites, that were decreased in adolescents with MDD but increased after treatment. While triglyceride abnormalities have been more frequently reported in depression, the role of bile acids remains less well characterized. Emerging evidence indicates that bile acids may act not only in cholesterol metabolism and lipid absorption, but also as signaling molecules involved in gut–brain axis regulation and mood disorders [34]. Existing studies suggest that levels of primary bile acids (such as CDCA) are significantly reduced in patients with depression, while the proportions of some secondary bile acids are abnormally elevated, which may reflect disturbances in the gut microbiota and its metabolic functions [37, 38]. Consistent with this view, the upregulation of bile acid‐related metabolites after fluoxetine treatment may reflect treatment‐associated remodeling of the gut–liver–brain metabolic network. Combined with clinical scoring data, the results of the Mantel Test analysis suggest that TG and FFA are highly correlated with the severity of MDD as reflected by the HAMA and CDRS‐R scales. Overall, these results suggest that plasma lipidomic remodeling may represent a peripheral biological feature associated with fluoxetine response in adolescent MDD. Further drug–lipid target and pathway enrichment analyses suggested that fluoxetine‐associated lipid changes may involve multiple biological processes, including neurotransmitter receptor signaling, inflammatory lipid mediator pathways, and endocrine or nuclear receptor‐related regulation. The enrichment of neuroactive ligand–receptor interaction was consistent with the known pharmacological action of fluoxetine on serotonergic neurotransmission [39]. In addition, pathways related to inflammatory mediator regulation of TRP channels and arachidonic acid metabolism suggest that changes in inflammatory lipid mediators may be involved in the lipidomic response to treatment [40, 41]. Several targets identified in the network, including PTGS2, ALOX5, LTA4H, PLA2G2A, and PLA2G5, are related to arachidonic acid metabolism, while nuclear receptor‐ and hormone‐related targets such as PPARA, RXRA, and VDR are closely linked to lipid metabolism and energy homeostasis [42, 43, 44, 45]. Combined with the observed treatment‐associated changes in triglycerides and bile acid‐related metabolites, these findings suggest that fluoxetine response in adolescent MDD may involve coordinated changes in neurotransmitter signaling, lipid inflammatory pathways, and peripheral lipid metabolism. This integrative analysis provides a potential biological framework linking fluoxetine treatment, lipid metabolic remodeling, and symptom improvement in adolescent MDD.

Although this study systematically analyzed the plasma lipidomic profiles of adolescents with MDD and the dynamic changes before and after fluoxetine treatment, certain limitations remain. First, this study was a single‐center cohort study; larger, multicenter cohorts are needed in the future to validate the generalizability of these findings. In addition, the absence of a placebo or untreated control limits attribution of the observed lipid changes specifically to fluoxetine. Second, while plasma samples were used for lipidomic analysis—which can reflect peripheral lipid metabolism—changes in cerebrospinal fluid lipids were not directly measured. Consequently, inferences regarding the specific mechanisms of lipid metabolism in the central nervous system remain indirect. Third, pubertal stage, sex hormones, menstrual status, diet, physical activity, and other metabolic factors were not systematically available and may therefore represent residual confounders. Finally, the analysis in this study was primarily based on statistical and machine learning models. Direct causal relationships between lipids and specific targets have not yet been experimentally validated. Further elucidation of these mechanisms through in vitro experiments and in vivo animal models is required in the future.

## 5 Conclusion

This study demonstrated disturbances of plasma lipid homeostasis in adolescents with MDD and identified several lipidomic signatures with potential discriminative value. Longitudinal lipidomic analysis showed that plasma lipid profiles changed following treatment with fluoxetine, suggesting that peripheral lipid metabolism may reflect treatment‐related biological changes. These findings provide new evidence of lipid metabolic abnormalities in adolescent MDD and may serve as a reference for future biomarker discovery and the identification of therapeutic targets.

## Author Contributions

C.L., Y.L., and H.H. contributed equally to this work. T.T., T.Q., and X.Z. jointly supervised the study. C.L., Y.L., H.H., T.T., T.Q., and X.Z. contributed to the conception and design of the study. C.L., Y.L., and H.H. performed the main statistical and bioinformatic analyses, interpreted the lipidomic data, prepared the figures and tables, and drafted the manuscript. J.G., B.Q., X.X., Y.H., and T.L. contributed to participant recruitment, clinical assessment, follow‐up evaluation, and biological sample collection. F.C., L.Z., Q.L., J.S., and H.X. contributed to sample processing, lipidomic data curation, literature review, and interpretation of the results. T.T., T.Q., and X.Z. obtained funding, supervised the project, and critically revised the manuscript for important intellectual content. All authors read and approved the final manuscript.

## Funding

This work was supported by STI2030‐Major Projects (2022ZD0212900), National Key R&D Program of China (2024YFC2707800), the National Natural Science Foundation of China (82271565, 82301714, 82301713), New Chongqing Youth Innovation Talent Project (CSTB2025YITP‐QCRCX0084), Chongqing Medical Scientific Research Project: Joint Project of Chongqing Health Commission and Science and Technology Bureau (2026QNKX006).

## Ethics Statement

This study was approved by the Institutional Review Board/Ethics Committee of the First Affiliated Hospital of Chongqing Medical University (Approval Nos. 2023–011‐02 and 20225402) and was conducted in accordance with the Declaration of Helsinki.

## Consent

Written informed consent was obtained from all participants and at least one parent or legal guardian before study participation.

## Conflicts of Interest

The authors declare no conflicts of interest.

## Supporting information


**Table S1:** Baseline demographic and clinical characteristics of the Longitudinally included and Longitudinally non‐included subgroups.
**Table S2:** Lipid species significantly altered after 8 weeks of fluoxetine treatment following FDR correction.
**Table S3:** Performance metrics of the five machine‐learning models based on pooled out‐of‐fold predictions. Accuracy, sensitivity, and specificity are reported for logistic regression (LR), random forest (RF), support vector machine (SVM), k‐nearest neighbors (KNN), and naive Bayes (NB).
**Table S4:** Baseline lipidomic comparison between the longitudinally included and longitudinally non‐included subgroups.
**Figure S1:** Participant flow diagram for the longitudinal lipidomic analysis. A total of 75 adolescents with major depressive disorder (MDD) comprised the cross‐sectional cohort. Of these, 44 completed 8 weeks of standardized fluoxetine monotherapy and were included in the longitudinal analysis, whereas paired week‐8 plasma samples were unavailable for the remaining 31 participants. MDD, major depressive disorder.
**Figure S2:** Distribution of detected plasma lipid species by lipid category and subclass. The donut chart shows the relative proportions of broad lipid categories and secondary lipid subclasses detected in the plasma lipidomic profiles of the study cohort.
**Figure S3:** Receiver operating characteristic (ROC) curves of the five machine‐learning models in the training folds, including logistic regression (LR), random forest (RF), support vector machine (SVM), naive Bayes (NB), and k‐nearest neighbors (KNN).
**Figure S4:** Association between the change in HexCer (d18:2/24:1) abundance and improvement in depressive symptoms after 8 weeks of fluoxetine treatment. Spearman correlation analysis was performed between Δlipid (week 8 − baseline) and ΔCDRS‐R (baseline − week 8).
**Figure S5:** Changes in HexCer(d18:2/24:1) and MG(16:0) according to treatment response.

## Data Availability

The data that support the findings of this study are available from the corresponding author upon reasonable request.
