# Supplementary material for: Plasma Lipidomic Features of Adolescent Major Depressive Disorder and Lipid Changes After Fluoxetine Treatment
Source: CNS Neurosci Ther. 2026 Oct 29;32(11):e71202. doi: 10.1002/cns.71202 (PMC13718314; doi:10.1002/cns.71202)
Supplement: Supplementary file 1 [file CNS-32-e71202-s001.docx]

**Supplementary Table 1**

Baseline demographic and clinical characteristics of the Longitudinally included and Longitudinally non-included subgroups.

|  | Longitudinally included | Longitudinally non-included | P |
| --- | --- | --- | --- |
| N | 44 | 31 |  |
| Sex (Female, Percentage)^a^ | 30 | 26 | 0.124 |
| Age (years, mean ± SD)^b^ | 15.86 ± 1.49 | 15.19 ± 1.62 | 0.068 |
| BMI (kg/m^2^, mean ± SD)^b^ | 20.60 ± 2.67 | 20.50 ± 2.66 | 0.874 |
| CDRS-R (mean ± SD)^b^ | 56.61 ± 9.48 | 58.77 ± 10.46 | 0.355 |
| HAMA (mean ± SD)^b^ | 17.40 ± 4.43 | 16.10 ± 3.86 | 0.192 |

Continuous variables are presented as mean ± standard deviation (SD); BMI, Body Mass Index; CDRS-R, Children’s Depression Rating Scale-Revised; HAMA, Hamilton Anxiety Rating Scale. a. Chi-Square test; b. T test.

**Supplementary Table 2**

Lipid species significantly altered after 8 weeks of fluoxetine treatment following FDR correction.

| **Compound** | **logFC** | **log_10_FC** | **AveExpr** | **t** | **P.Value** | **FDR** | **B** | **VIP** |
| --- | --- | --- | --- | --- | --- | --- | --- | --- |
| FFA(10:0) | -74.2123 | -22.3401 | 37.9136 | -11.3270 | 9.12E-19 | 7.89E-16 | 32.1952 | 4.7346 |
| Glycocholic acid | 0.2759 | 0.0831 | 0.4368 | 4.1958 | 0.00007 | 0.00726 | 0.7639 | 2.8258 |
| HexCer(t18:1/20:1(2OH)) | -0.6012 | -0.1810 | 3.1660 | -4.0448 | 0.00011 | 0.01090 | 0.2406 | 3.0635 |
| MG(16:0) | -6.0357 | -1.8169 | 64.6185 | -3.8951 | 0.00019 | 0.01517 | -0.2653 | 2.7680 |
| TxB3 | -0.5235 | -0.1576 | 0.7561 | -3.8330 | 0.00023 | 0.01641 | -0.4712 | 2.5602 |
| DG(16:0_16:0) | -0.5579 | -0.1679 | 4.6440 | -3.8247 | 0.00025 | 0.01641 | -0.4984 | 2.5057 |
| FFA(16:0) | -59.1182 | -17.7964 | 316.1589 | -3.5419 | 0.00064 | 0.03707 | -1.4043 | 2.3005 |
| HexCer(t14:1/24:1(2OH)) | -0.2943 | -0.0886 | 1.7264 | -3.4169 | 0.00097 | 0.04923 | -1.7884 | 2.7125 |

**S****upplementary Table 3. Performance metrics of the five machine-learning models based on pooled out-of-fold predictions.** Accuracy, sensitivity, and specificity are reported for logistic regression (LR), random forest (RF), support vector machine (SVM), k-nearest neighbors (KNN), and naive Bayes (NB).

| **Model** | **Pooled CV AUC** | **AUC CI low** | **AUC CI mid** | **AUC CI high** | **Pooled CV Accuracy** | **Pooled CV Sensitivity** | **Pooled CV Specificity** |
| --- | --- | --- | --- | --- | --- | --- | --- |
| LR | 0.852 | 0.791 | 0.852 | 0.913 | 0.760 | 0.747 | 0.773 |
| RF | 0.843 | 0.783 | 0.843 | 0.904 | 0.740 | 0.760 | 0.720 |
| SVM | 0.821 | 0.753 | 0.821 | 0.889 | 0.747 | 0.707 | 0.787 |
| KNN | 0.791 | 0.718 | 0.791 | 0.864 | 0.727 | 0.840 | 0.613 |
| NB | 0.789 | 0.717 | 0.789 | 0.862 | 0.713 | 0.787 | 0.640 |

**Supplementary Table 4.** Baseline lipidomic comparison between the longitudinally included and longitudinally non-included subgroups.

| **Compound** | **log_10_FC** | **AveExpr** | **t** | **P.Value** | **FDR** | **B** | **VIP** |
| --- | --- | --- | --- | --- | --- | --- | --- |
| FFA(10:0) | -10.1280 | 61.1134 | -3.0357 | 0.0033 | 0.9886 | -2.7566 | 2.2998 |
| TxB3 | -0.1049 | 0.8738 | -2.3156 | 0.0234 | 0.9886 | -4.5213 | 1.1782 |
| LPE(0:0/16:0) | 0.0836 | 1.6838 | 2.2147 | 0.0299 | 0.9886 | -4.7364 | 2.5050 |
| PMeOH(16:0_16:0) | 0.0812 | 9.6925 | 2.1983 | 0.0311 | 0.9886 | -4.7704 | 1.3546 |
| LPE(18:2/0:0) | 0.2792 | 3.2427 | 2.1600 | 0.0340 | 0.9886 | -4.8494 | 3.1422 |
| LPE(16:0/0:0) | 0.0812 | 1.6697 | 2.1297 | 0.0365 | 0.9886 | -4.9111 | 2.3363 |
| LPE(0:0/18:2) | 0.2696 | 3.2645 | 2.0270 | 0.0463 | 0.9886 | -5.1141 | 2.1772 |

**Supplementary Figure 1. Participant flow diagram for the longitudinal lipidomic analysis.** A total of 75 adolescents with major depressive disorder (MDD) comprised the cross-sectional cohort. Of these, 44 completed 8 weeks of standardized fluoxetine monotherapy and were included in the longitudinal analysis, whereas paired week-8 plasma samples were unavailable for the remaining 31 participants. MDD, major depressive disorder.

**Supplementary Figure 2.** Distribution of detected plasma lipid species by lipid category and subclass. The donut chart shows the relative proportions of broad lipid categories and secondary lipid subclasses detected in the plasma lipidomic profiles of the study cohort.

**Supplementary Figure 3.** Receiver operating characteristic (ROC) curves of the five machine-learning models in the training folds, including logistic regression (LR), random forest (RF), support vector machine (SVM), naive Bayes (NB), and k-nearest neighbors (KNN)**.**

**Supplementary Figure 4**. Association between the change in HexCer(d18:2/24:1) abundance and improvement in depressive symptoms after 8 weeks of fluoxetine treatment. Spearman correlation analysis was performed between Δlipid (week 8 − baseline) and ΔCDRS-R (baseline − week 8).

**Supplementary Figure 5.** Changes in HexCer(d18:2/24:1) and MG(16:0) according to treatment response.

(A)AHexCer(d18:2/24:1) and (B) AMG(16:0) in responders and non-responders. Alipid was defined as week 8 minus baseline, and treatment response was defined as a ≥50% reduction in CDRS-R score from baseline. Differences between groups were evaluated using limma.
